# Investigation of Shape Memory Polyurethane Properties in Cold Programming Process Towards Its Applications

**DOI:** 10.3390/polym16020219

**Published:** 2024-01-12

**Authors:** Maria Staszczak, Leszek Urbański, Mariana Cristea, Daniela Ionita, Elżbieta Alicja Pieczyska

**Affiliations:** 1 Institute of Fundamental Technological Research, Polish Academy of Sciences, 02-106 Warsaw, Poland; mstasz@ippt.pan.pl (M.S.); lurban@ippt.pan.pl (L.U.); 2 “Petru Poni” Institute of Macromolecular Chemistry, 700487 Iasi, Romania; mcristea@icmpp.ro (M.C.); ionita.daniela@icmpp.ro (D.I.)

**Keywords:** polyurethane shape memory polymer, cold programming, thermal expansion, shape fixity, shape recovery

## Abstract

Thermoresponsive shape memory polymers (SMPs) with the remarkable ability to remember a temporary shape and recover their original one using temperature have been gaining more and more attention in a wide range of applications. Traditionally, SMPs are investigated using a method named often “hot-programming”, since they are heated above their glass transition temperature (*T_g_*) and after that, reshaped and cooled below *T_g_* to achieve and fix the desired configuration. Upon reheating, these materials return to their original shape. However, the heating of SMPs above their *T_g_* during a thermomechanical cycle to trigger a change in their shape creates a temperature gradient within the material structure and causes significant thermal expansion of the polymer sample resulting in a reduction in its shape recovery property. These phenomena, in turn, limit the application fields of SMPs, in which fast actuation, dimensional stability and low thermal expansion coefficient are crucial. This paper aims at a comprehensive experimental investigation of thermoplastic polyurethane shape memory polymer (PU-SMP) using the cold programming approach, in which the deformation of the SMP into the programmed shape is conducted at temperatures below *T_g_*. The PU-SMP glass transition temperature equals approximately 65 °C. Structural, mechanical and thermomechanical characterization was performed, and the results on the identification of functional properties of PU-SMPs in quite a large strain range beyond yield limit were obtained. The average shape fixity ratio of the PU-SMP at room temperature programming was found to be approximately 90%, while the average shape fixity ratio at 45 °C (*T_g_* − 20 °C) was approximately 97%. Whereas, the average shape recovery ratio was 93% at room temperature programming and it was equal to approximately 90% at 45 °C. However, the results obtained using the traditional method, the so-called hot programming at 65 °C, indicate a higher shape fixity value of 98%, but a lower shape recovery of 90%. Thus, the obtained results confirmed good shape memory properties of the PU-SMPs at a large strain range at various temperatures. Furthermore, the experiments conducted at both temperatures below *T_g_* demonstrated that cold programming can be successfully applied to PU-SMPs with a relatively high *T_g_*. Knowledge of the PU-SMP shape memory and shape fixity properties, estimated without risk of material degradation, caused by heating above *T_g_*, makes them attractive for various applications, e.g., in electronic components, aircraft or aerospace structures.

## 1. Introduction

Shape memory polymers (SMPs) are gaining increasing attention due to their versatile functionality [1,2,3,4]. These innovative materials exhibit the capability to retain a temporary shape and subsequently return to their initial shape upon exposure to an external stimulus, e.g., heat [1,5,6,7], moisture [6,8,9], electric field [10], magnetic field [11], pH [12,13], microwaves [14], or light [15,16]. Thermoresponsive shape memory polymers with the remarkable ability to remember their temporary shape and recover their original one through temperature are the largest class of SMPs [2,3,5,17]. The activation temperature of a thermoresponsive SMP is usually determined by its glass transition temperature *T_g_*. At this temperature, polymers undergo a transition from a glassy state with frozen molecular mobility to a rubbery state with increased molecular mobility accompanied by changes in physical and mechanical properties [18]. For example, the polymer exhibits a high elastic modulus below the *T_g_*, while above the *T_g_*, the modulus significantly decreases, enabling the release of internal stresses and facilitating the shape recovery process. Therefore, the *T_g_* is a key parameter that defines the polymer’s behavior because it influences the different molecular structures of the polymer segments, depending on whether they are below or above this particular temperature [2,5].

Shape memory properties that define the ability of the polymer to fix the temporary shape and to recover the original one are investigated in the thermomechanical loading cycle with respect to the SMP glass transition temperature. Traditionally, SMPs’ shape memory properties are investigated using a method called “hot-programming” which consists in heating the polymers above *T_g_*, followed by loading, cooling, unloading and reheating [19]. Usually, the researchers involved in research on SMPs use the traditional hot programming technique in order to characterize the shape memory behavior of SMPs [7,17,19,20]. However, this method has some disadvantages. The initial heating step during the shape memory polymer cycle to trigger the change of the SMP shape enables it to reach a rubbery state and to be deformed easily. This time-consuming process also creates a temperature gradient within the material structure and causes significant thermal expansion of the polymer sample resulting in a reduction in its shape recovery [21]. This, in turn, limits the application fields of shape memory polymers, in which fast actuation, dimensional stability and low thermal expansion coefficient are crucial. Moreover, typically, the process entails heating the entire SMP specimen, deforming it, and subsequently cooling it while retaining the altered shape. This method results in increased overall energy consumption and creates significant challenges for local programming.

Among thermally activated SMPs, polyurethane shape memory polymers (PU-SMPs) are distinguished because they are characterized by a wide range of shape recovery temperatures, good processability, biocompatibility and deformability, as well as being a high recoverable strain, resulting in favorable functional and mechanical properties [1,5,7,22,23]. Thermoplastic shape memory polyurethanes have a linear molecular structure, which is physically crosslinked through physical intermolecular interactions. PU-SMPs are multi-block copolymers composed of hard and soft segments arranged randomly, forming a two-phase structure. Hard segments typically exhibit higher transition temperatures, indicating the temperature at which polymeric chains gain mobility compared to soft segments. This intrinsic incompatibility between the segments prompts the separation of these two blocks and leads to microphase separation, ultimately inducing the shape memory effect [24]. The interplay between hard and soft segments, whether below or above *T_g_*, is crucial for locking in the temporarily deformed shape and restoring the original one. The hard segments function as act points consisting of physical crosslinks to stabilize the original shape. In contrast, the soft segments act as molecular switches allowing for the fixation of the temporary shape, as well as the shape recovery in proper conditions [1,2,7].

The PU-SMP properties discussed above, combined with a relatively high strength, as for polymers, and their ability to undergo reversible shape changes in response to temperature, make them valuable materials in various modern industries. They find applications in biomedical devices such as self-expandable vascular stents [25,26], drug delivery systems [11,27,28], eyeglasses frames and orthodontic appliances [29], in robotics and smart actuators [30,31], and in aircraft and aerospace industries [32,33]. Other directions of PU-SMP applications are smart textiles and thermo-responsive clothes [34,35,36,37,38]. PU-SMPs are also applied in consumer goods, such as shoe insoles [34,39] and responsible packages for valuable products, or smart foils for food protection, where the SMP smart structure enables the exchange of both heat and humidity [40].

PU-SMPs often exist in their glassy states at room temperature. Therefore, they can be programmed through a cold programming (CP) approach which allows them to achieve a new shape at a temperature below *T_g_*. Firstly, in the CP process, a PU-SMP undergoes deformation beyond its yield point at a temperature below the *T_g_*. Next, upon unloading, there is a small instantaneous spring back, releasing some of the stored energy. Then, PU-SMP structural relaxation occurs as the SMP undergoes viscoelastic recovery. The final step involves restoring the original shape by heating the PU-SMP above its glass transition temperature [41,42]. The attractiveness of CP lies in its ability to save both time and energy through isothermal programming at room temperature or other temperatures below *T_g_*. However, it should be noted that CP requires deformation to occur in the plastic zone beyond the yield limit. Without meeting this criterion, the PU-SMP would return to its original shape [43,44,45,46].

To date, cold programming has not been widely studied. However, some works have been published. Li et al. investigated the shape fixity and shape recovery properties of polystyrene-based thermoset SMPs in cold compression, showing this method as efficient and allowing high shape fixity to be achieved [44]. Lewis et al. studied the effect of cold drawing on the shape memory properties of polycaprolactone (PCL)-based networks [47]. They demonstrated that the extent of shape fixity during cold drawing is associated with the level of strain-induced crystallization. Abishera et al. investigated the so-called “reversible plasticity shape memory (RPSM)” effect, which occurs during CP, in pure epoxy-based SMPs and epoxy-based SMPs reinforced with multi-walled carbon nanotubes (MWCNT) [48]. The sample was plastically deformed at room temperature (below *T_g_*) and the temporary shape was obtained, whereas during subsequent heating to a temperature above *T_g_*, the original shape was restored. With the increase in MWCNT content, shape memory properties were improved. Bhattacharya et al. also studied the RPSM effect in a polymer blend consisting of a soft thermoplastic polyurethane (TPU) and a low melting temperature thermoplastic healing agent—PCL [49]. They showed quite a high shape fixity of 88% for the TPU/PCL blend containing 50 wt % TPU and 50 wt % PCL, compared to that of pure TPU which was 16%. Shani et al. experimentally examined the shape memory properties of thermoset epoxy-based SMPs in cold uniaxial tensile loading [42].

To the best of the authors’ knowledge, the shape memory properties of thermoplastic PU-SMPs have not been investigated, in particular, with a higher value of *T_g_* (65 °C), during cold drawing. Therefore, as a result of the growing use of multifunctional polyurethanes in various fields, the shape memory properties of PU-SMPs, which can be activated at room temperature, require further investigation.

The proposed work concerns a comprehensive experimental investigation of thermoplastic polyurethane shape memory polymers (PU-SMPs) using the cold programming approach. Structural, mechanical and shape memory characterization has been conducted. The effect of loading temperature on the mechanical behavior of PU-SMPs during tension in the thermal chamber has been investigated. PU-SMP functional properties, i.e., shape fixity and shape recovery parameters in the cold programming approach have been determined with high accuracy. The influence of the PU-SMP deformation temperature on the cold programming process was also studied. To this end, two temperatures were chosen, room temperature 25 °C and 45 °C, as temperatures close to the glass transition region. Moreover, the shape memory properties have been investigated in the conventional hot programming (HP) approach at 65 °C and compared with those obtained in cold programming.

## 2. Materials and Methods

### 2.1. Materials and Specimens

**Figure 1:**
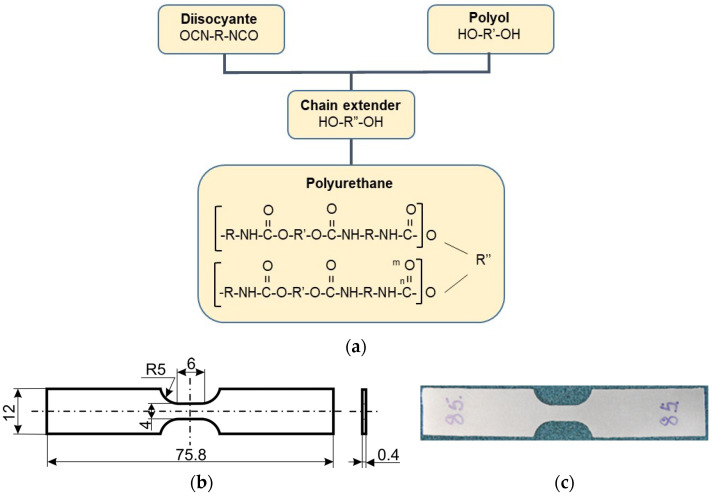
(**a**) Basic synthesis process of polyurethane shape memory polymer PU-SMP; (**b**) shape and dimensions and (**c**) photograph of dog-bone specimen designed for mechanical and thermomechanical testing.

The material used in this research is a thermoplastic polyurethane shape memory polymer manufactured by SMP Technologies Inc., Tokyo, Japan, characterized by a glass transition temperature of approximately 65 °C and denoted by the producer as MM6520. The basic synthesis process of this material is shown in Figure 1a [1,22]. The shape and dimensions of the PU-SMP specimen designed after numerous trials and discussions for the experimental investigation of mechanical and thermomechanical properties are presented in Figure 1b, while its photograph is in Figure 1c, respectively.

In order to relax the internal stresses and uniform the structure, the PU-SMP specimens were heated up to 80 °C (*T_g_* + 15 °C) for 30 min before mechanical and thermomechanical testing. After that, the specimens were taken out from the thermal chamber and cooled down in room conditions. This procedure allowed us to erase the thermal history of the polymer.

### 2.2. Differential Scanning Calorimetry (DSC) and Modulated Differential Scanning Calorimetry (MDSC)

The thermal response of the PU-SMP was characterized using differential scanning calorimetry (DSC) analysis by a Discovery DSC 250 (TA Instruments, New Castle, DE, USA) under a N_2_ atmosphere. Enthalpy (cell) constant calibration was performed with high purity indium. The PU-SMP sample with a weight of 5.9 mg was sealed in standard aluminum pans. The heating–cooling–heating DSC run was performed between −100 °C and 200 °C, with a heating rate of 20 °C/min.

Another approach to determine the glass transition temperature from the first heating of the DSC experiment was modulated differential scanning calorimetry (MDSC). MDSC separates the total heat flow of DSC into two parts based on the heat flow that does and does not respond to a changing heating rate. MDSC analysis was also carried out using a Discovery DSC 250 (TA Instruments, New Castle, DE, USA) under a N_2_ atmosphere in a temperature range from −50 °C to 200 °C. However, the heating rate was lower than in the conventional DSC and equal to 3 °C/min.

### 2.3. Dynamic Mechanical Analysis (DMA)

The viscoelastic properties of PU-SMP were characterized by conducting dynamic mechanical analysis (DMA). To this end, a Perkin Elmer Diamond DMA instrument (Waltham, MA, USA) was used. Single-frequency DMA experiments were conducted in tension mode, on PU-SMP films with a length of 10 mm, width of 10 mm and thickness of 0.4 mm. The single-frequency temperature test was run by increasing the temperature in ramp mode at a heating rate of 2 °C/min and a frequency of 1 Hz in a temperature range from −150 °C to 150 °C. The PU-SMP samples’ behavior was investigated in a longitudinal direction to the loading direction.

The multifrequency experiments were performed to establish the nature of transition (relaxation or melting/crystallization). These experiments were conducted at five frequencies (0.5, 1, 2, 5, 10 Hz) using the same instrument, also in tension ramp mode, with 2 °C/min, in the same temperature range. Moreover, single-frequency DMA was repeated after a multifrequency experiment.

### 2.4. The Mechanical and Thermomechanical Investigation of PU-SMP

**Figure 2:**
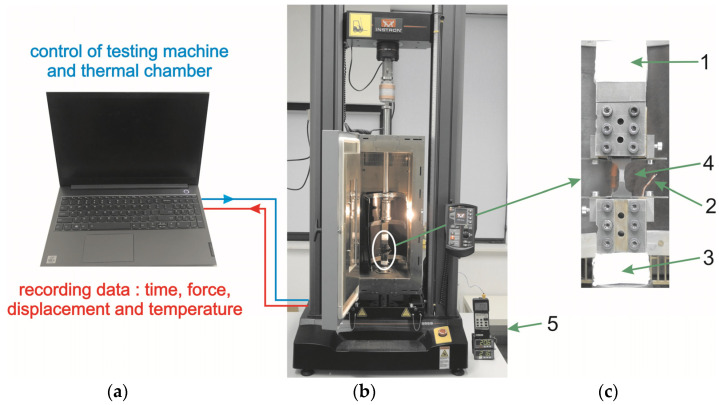
Schematic diagram of the used testing setup: (**a**) computer, (**b**) testing machine with the thermal chamber, (**c**) specimen in the grips of the testing machine showing thermocouples 1—on the upper grip, 2—in specimen area, 3—on the lower grip, 4—specimen, 5—temperature indicator.

The mechanical investigation of the PU-SMP was carried out on the Instron 5969 testing machine (Instron, Norwood, MA, USA) coupled to the Instron thermal chamber 3119-606 (Figure 2b). Generally, the temperature in the thermal chamber is measured using only one thermocouple, located near the air inlet, as designed by the producer. The authors have also introduced three extra thermocouples into the thermal chamber to uniform the temperature and enhance the experiment’s precision. The first thermocouple was positioned on the upper grip of the testing machine (1), the second in proximity to the specimen gauge length (2), and the third on the lower grip (3), respectively (Figure 2c). Once the desired temperature was set in the chamber, the temperature of each thermocouple was monitored. The PU-SMP specimen loading started only when the temperature variation between the three thermocouples was less than 1 °C, ensuring a specimen temperature measurement accuracy within ±1 °C.

The experiments began with tension tests performed with a strain rate of 10^−3^ s^−1^ until rupture (see Section 3.2) in order to characterize the PU-SMPs’ mechanical behavior and to choose an appropriate deformation range for the thermomechanical loading program. These experiments were conducted in isothermal conditions at two temperatures below the glass transition region: at room temperature (about 25 °C) and at 45 °C (*T_g_* – 20 °C).

The stress and strain values presented in all mechanical curves are related to the instantaneous values of the specimen cross-section, obtaining the so-called “true stress” and “true strain” values [50]. This gives more accurate results in the case of large deformation when the cross-section changes significantly.

The shape memory properties of PU-SMPs were investigated using a thermomechanical loading program in the cold programming approach, in which the deformation of the SMPs into the programmed shape was conducted at temperatures below *T*_g_. In addition, the shape fixity and shape recovery values were determined through a traditional hot programming cycle conducted at the *T_g_* of the PU-SMP. More experimental details are presented in Section 3.3, since the detailed procedure presented just before the experimental results will help readers properly understand this rather complicated research program.

## 3. Results and Discussion

### 3.1. Results and Analysis of the Structural Characterization of PU-SMP T_g_ = 65 °C

#### 3.1.1. Differential Scanning Calorimetry and Modulated Differential Scanning Calorimetry

**Figure 3:**
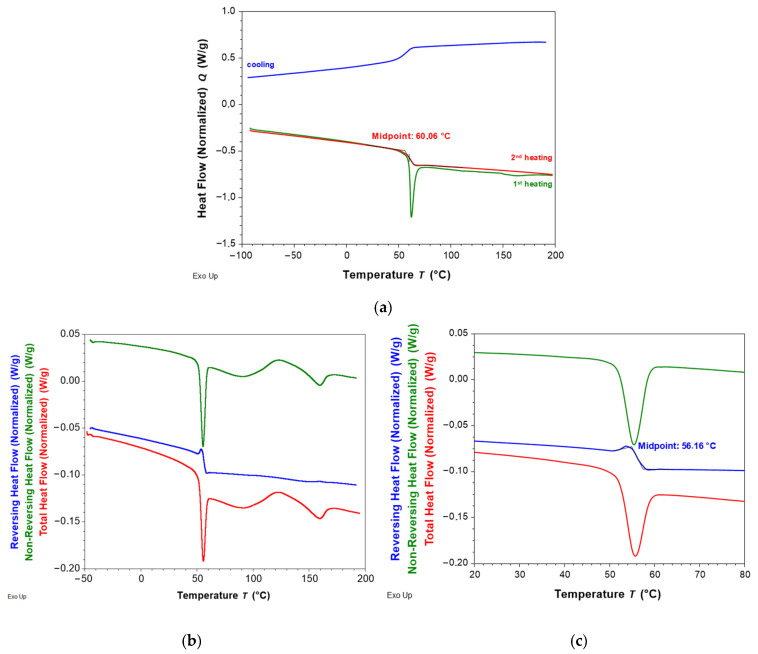
Results obtained for PU-SMP samples of: (**a**) conventional DSC; (**b**) MDSC in a temperature range from −50 °C to 200 °C and (**c**) detail for the MDSC experiment in a temperature range from 20 °C to 80 °C.

The obtained results of the conventional heating–cooling–heating DSC experiment conducted for the PU-SMP are presented in Figure 3a. The glass transition temperature value cannot be determined from the first heating scan due to the presence of overlapping events. *T_g_* is observed in the DSC curve as a step change in heat flow, not as a peak. In this case, *T_g_* was determined from the second heating scan and equaled approximately 60 °C.

Another approach that enables determining the glass transition temperature from the first heating of the DSC experiment is to use modulated differential scanning calorimetry. The obtained MDSC results for the PU-SMP in a temperature range from −50 °C to 200 °C are shown in Figure 3b, while the use of a smaller temperature range for better visualization from 20 °C to 80 °C is shown in Figure 3c. It shows the crystallization that is followed by melting on the non-reversible heat flow curve. The reversing heat flow signal shows a glass transition near 56.2 °C. The *T_g_* shifts to a lower temperature compared to the conventional DSC experiment due to the lower heating rate used in the MDSC technique (3 °C/min).

#### 3.1.2. Dynamic Mechanical Analysis Results

**Figure 4:**
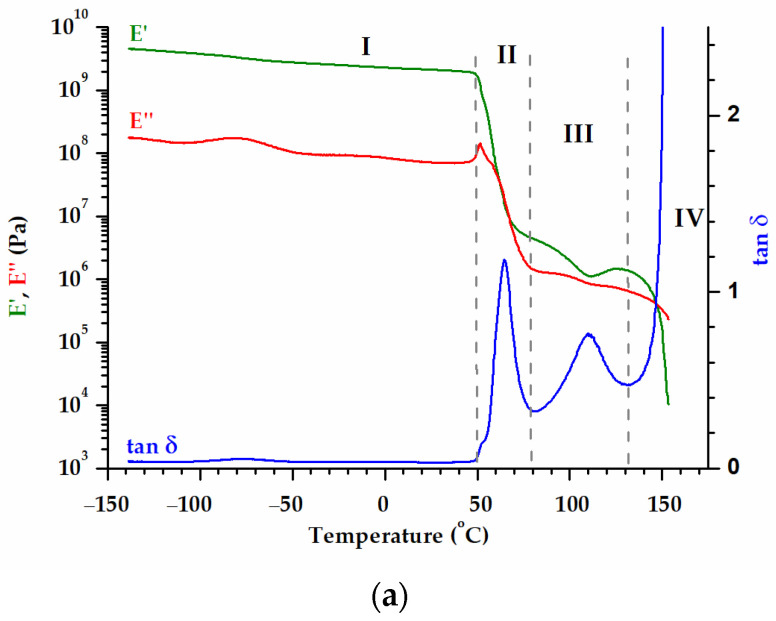

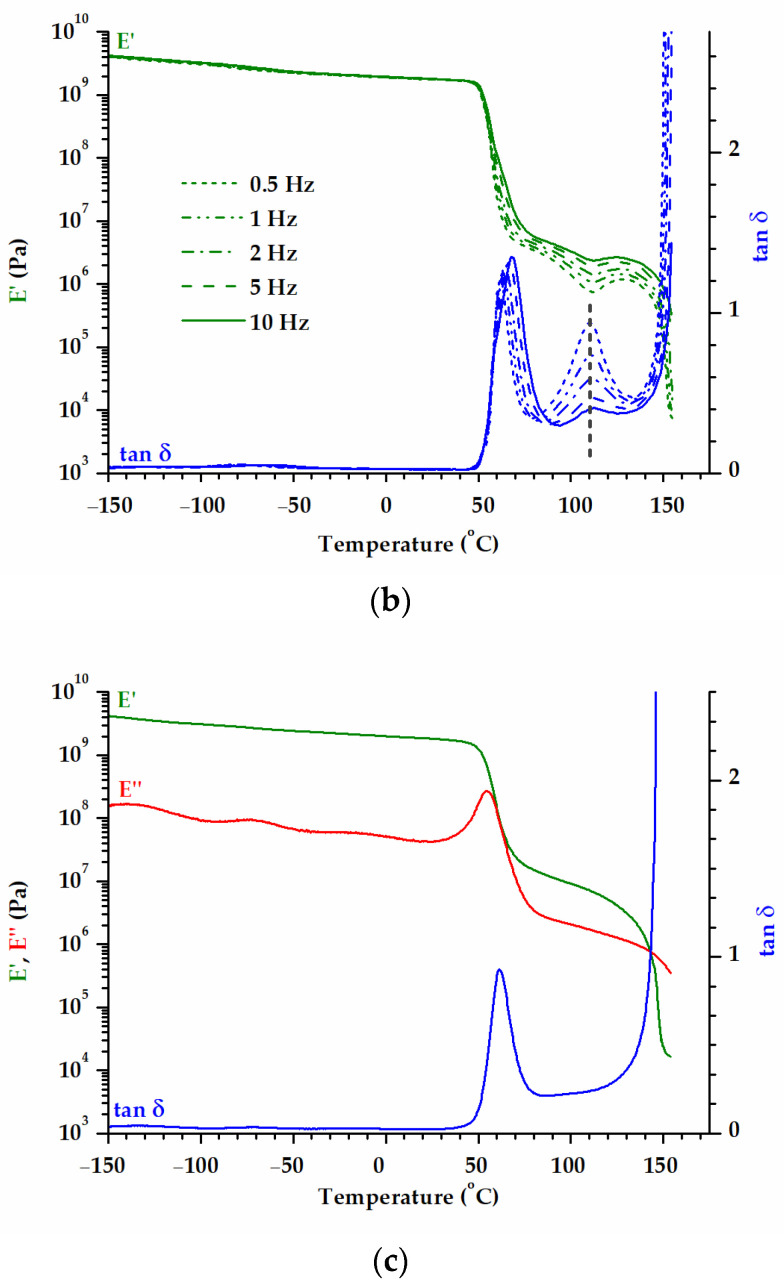
Variation in storage modulus *E*′ (green color), loss modulus *E*″ (red color) and loss factor tan *δ* (blue color) with temperature obtained for the PU-SMP in (**a**) conventional DMA experiment, (**b**) multifrequency experiment (*E*″ curves were not included in this figure making it difficult to distinguish between numerous lines) and (**c**) after multifrequency experiment.

The storage modulus *E*′, loss modulus *E*″ and loss factor tan *δ* as a function of temperature obtained during DMA experiments conducted for the PU-SMP are presented in Figure 4. The behavior of the polymer changes with the temperature according to its molecular mobility. Considering the modulus behavior, four distinct regions in the diagram can be identified: the glassy region (I), the glass transition (II), the rubbery (III) and the flowing (IV) regions (Figure 4a). The drop in the *E*′ curve and the peak in the tan *δ* curve provide information about the glass transition temperature (*T_g_*) and the polymer behaviors at various temperatures (Figure 4, Table 1).

The *T_g_* determined as the peak of loss tangent tan *δ* is approximately equal to 65 °C, very close to the value provided by the producer.

Secondary relaxations have been observed for the PU-SMP in the negative temperature range. The glass transition is followed by a phenomenon that is frequency independent; considering the *E*′ trend in that region (100–150 °C, Figure 4b,c)—decreasing followed by increasing—it is very probable to have a succession of overlapping melting/crystallization phenomena. These phenomena are emphasized by the DSC (Figure 3).

### 3.2. Results and Analysis of Mechanical Characteristics of PU-SMP T_g_ = 65 °C at Various Temperatures

In order to learn more about the PU-SMP mechanical behavior in various conditions, tensile loadings were conducted for a strain rate of 10^−3^ s^−1^ at three various temperatures: two temperatures below *T_g_* (25 °C and 45 °C) until specimen rupture and at a temperature equal to *T_g_* (65 °C) until maximum possible strain value was obtained, avoiding damage of the Instron thermal chamber. To check the repeatability of measurements, three mechanical tests were performed at each temperature and similar curves’ behavior was observed.

**Figure 5:**
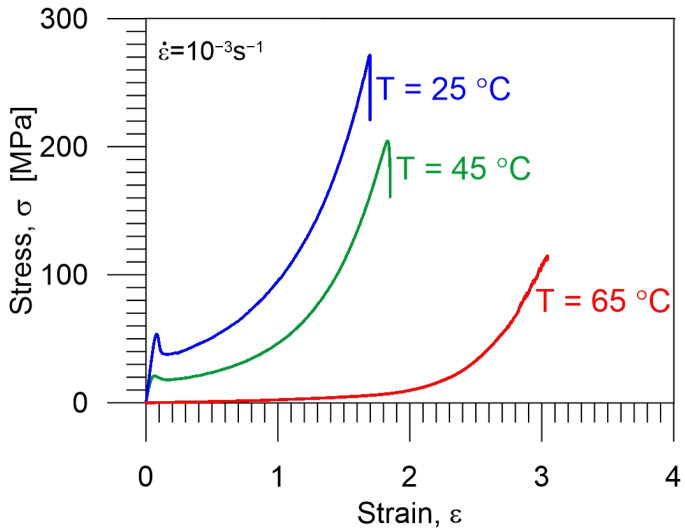
Comparison of mean mechanical curves obtained for PU-SMP *T_g_ =* 65 °C during tension until rupture with a strain rate of 10^−3^ s^−1^ at temperatures: 25 °C, 45 °C and 65 °C.

A comparison of the average results, i.e., stress–strain curves at 25 °C, 45 °C and 65 °C, is depicted in Figure 5. In the case of 65 °C, conducting the tension until rupture was not possible due to thermal chamber limitations. Namely, any of the three specimens were ruptured within circa 6 h during the experiment duration and the tests were automatically stopped by the preset displacement limits of the Instron testing machine system.

At temperatures below the glass transition temperature, the polymer was in a glassy state and behaved similarly to elasto-plastic material [5,22,32]. The stress–strain curves of all samples at both temperatures exhibit the following stages. The first stage is the reversible, linear region of the deformations, indicating an elastic response, which is described by the theory of solid elasticity. The strain is low and the specimen shape does not change significantly. The second is the plastic stage, associated with irreversible mechanisms of polymer deformation of a dissipative nature. At this stage, the linear segment reaches the maximum stress, marking the yield point, after which the stress decreases due to the strain localization phenomenon. Further, as the strain increases, the stress also becomes higher. The third stage is the damage stage, associated with the breaking of the polymer chains, leading to the specimen rupture [51,52,53].

As it can be noticed in Figure 5, the mechanical characteristics of PU-SMP at room temperature (25 °C) and at 45 °C are different from the characteristics at 65 °C (*T_g_* region). By comparing Young’s modulus of the PU-SMP (slope of the initial linear part of the stress–strain curves), it has been confirmed that the elastic modulus of the polymer is much higher when the specimen is deformed at a lower temperature (Table 2). The average value of yield strength is also higher for the lower temperature, as reported in [7]. At temperatures below *T_g_* the soft segments, which act as a reversible phase, do not have enough energy to obtain good mobility, and the lower the loading temperature, the higher the stress values the polymer needs to be deformed. On the other hand, the elongation at break is higher for the higher temperature (Figure 5, Table 2). The higher the temperature, the lower the stress and the higher the observed deformability of the polymer.

In the vicinity of or above *T_g_*, the PU-SMP enters a rubbery state, facilitating easy deformation. As a result, the stress values and elastic moduli recorded at *T_g_* are notably lower than those observed at 25 °C (*T_g_* – 40 °C) and 45 °C (*T_g_* – 20 °C), and a pronounced yield point is not observed. This can be explained by the fact that as the temperature approaches *T_g_*, a transition into a rubbery state occurs which activates the soft segment movements resulting in the polymer’s higher entropy, internal energy and flexibility of the polymer chains.

### 3.3. Characterization of PU-SMP T_g_ = 65 °C Shape Memory Properties

The shape memory properties of PU-SMP were investigated by a thermomechanical loading program. One of the directions of the research was studying the effect of programming temperature on the thermomechanical loading cycle. Shape memory cycles were performed for two different temperatures 25 °C and 45 °C, which are suitably below the glass transition temperature of PU-SMP (65 °C); however, the temperature of 45 °C is close to the *T_g_* region (see Figure 4a). Moreover, the obtained results were compared with those obtained in the traditional hot programming approach, which was performed at 65 °C.

#### 3.3.1. Investigation of PU-SMP *T_g_* = 65 °C Shape Memory Properties at Temperature of 25 °C

**Figure 6:**
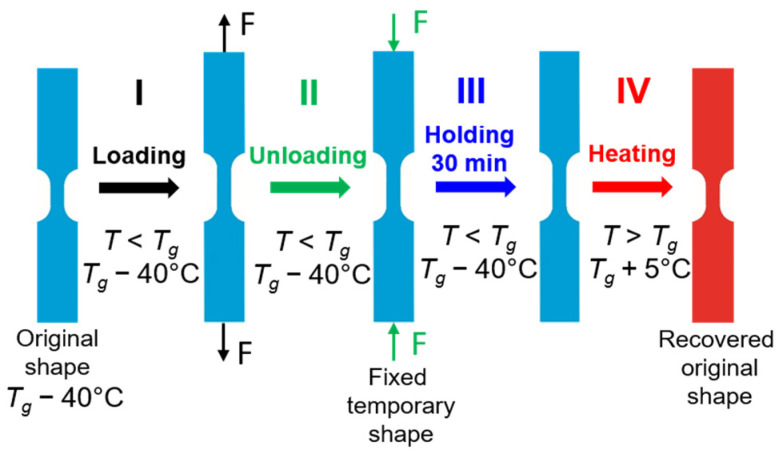
Schematic demonstration of subsequent stages (I–IV) of the thermomechanical loading program at *T_room_* = 25 °C presenting changes in shape and gauge length of the PU-SMP specimen.

The schematic demonstration of the thermomechanical loading program at room temperature *T_room_* = 25 °C in CP approach presenting the subsequent I–IV stages of the thermomechanical loading and showing the shape and gauge length changes in the specimens is depicted in Figure 6.

During step I, the specimen is mounted in the grips of the testing machine and deformed by tension at room temperature (25 °C) with a strain rate of 10^−3^ s^−1^ to a particular strain value beyond the yield limit (60%). In step II, the specimen is unloaded with the same strain rate to the zero-force value, which is accompanied by a small strain recovery, so-called “spring back”, which is a characteristic phenomenon for polymers. During step III, the specimen is held in this state for 30 min. The PU-SMP contraction occurs which is a result of the viscoelastic recovery of SMP under isothermal conditions. This phenomenon refers to PU-SMP structural relaxation which allows the material to stabilize its state [44]. Then, in step IV, the specimen is heated from *T_room_* to *T_g_ +* 5 °C = 70 °C with a rate of 12 °C/min under no-load conditions, leading to the recovery of the PU-SMP’s original shape.

**Figure 7:**
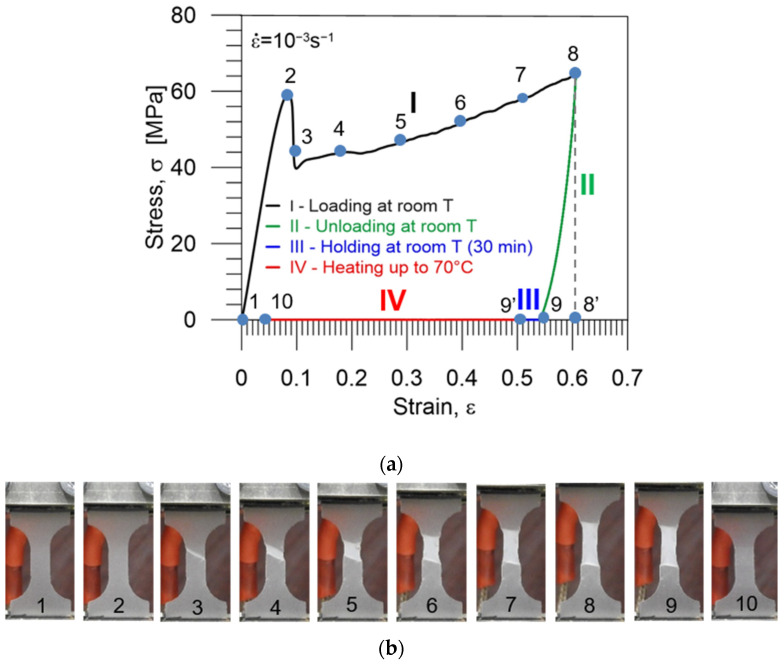
(**a**) Stress vs. strain curve obtained during the PU-SMP thermomechanical loading cycle at a loading temperature of 25 °C. Particular colors mark subsequent stages of the program: I (black)—loading at room temperature (25 °C), II (green)—unloading to zero stress at room temperature, III (blue)—holding at room temperature during 30 min (structural relaxation), IV (red)—heating up to 70 °C. Numbers 1–10 correspond to the strain values in thermomechanical loading cycle for which the photographs below were taken, while 8′ and 9′ denote the maximum strain value *ε_m_* and the strain after holding at room temperature *ε_h_*, respectively; (**b**) photographs showing subsequent stages of specimen deformation: 1—before loading, 2–8—during loading, 9—after unloading (shape fixity), 10—after heating to 70 °C (shape recovery).

An example of the experimental results, obtained during the cycle of thermomechanical loading in a cold programming approach at room temperature, i.e., the stress vs. strain, is presented in Figure 7a. Selected colors used in the diagrams indicate each stage of the program: I (black)—loading at room temperature (25 °C), II (green)—unloading to zero stress at room temperature, III (blue)—holding at room temperature during 30 min (structural relaxation), IV (red)—heating up to 70 °C.

The strain values at points 8′, 9, 9′ and 10 (Figure 7a) correspond to *ε_m_* which denotes the maximum strain value, to *ε_un_*—the strain obtained after unloading, to *ε_h_*—the strain after holding at room temperature and to *ε_ir_*—the irrecoverable strain obtained after heating up to *T_g_* + 5 °C under no-load conditions, respectively. 

Below the diagram, photographs taken at various strain values (marked in Figure 7a) showing the subsequent stages of the PU-SMP specimen deformation during the thermomechanical loading cycle are presented (Figure 7b): 1—before loading, 2—at the maximal achieved average stress, i.e., just before nucleation of the strain localization, 3—during further loading, 4–8—presenting subsequent stages, i.e., evolution of the strain localization, 9—after unloading (shape fixity), 10—after heating to 70 °C (*T_g_* + 5 °C) (shape recovery).

**Figure 8:**
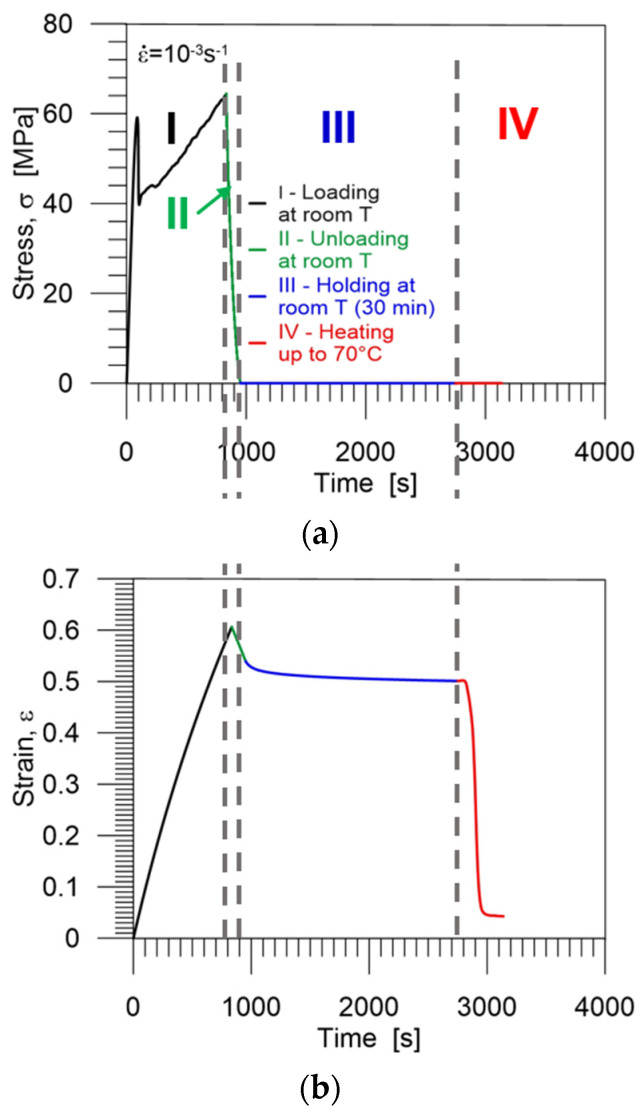
Stress (**a**) and strain (**b**) vs. time curves obtained during the PU-SMP thermomechanical loading cycle at 25 °C. Particular colors mark subsequent stages of the program: I (black)—loading at room temperature (25 °C), II (green)—unloading to zero stress at room temperature, III (blue)—holding at room temperature for 30 min, IV (red)—heating up to 70 °C (*T_g_* + 5 °C).

Diagrams demonstrating the procedure and showing the experimental results, obtained during the cycle of thermomechanical loading in the cold programming approach at 25 °C, i.e., the stress and strain vs. time are presented in Figure 8a and Figure 8b, respectively. The selected colors used in the diagrams indicate each stage of the program: I (black)—loading at room temperature (25 °C), II (green)—unloading to zero stress at room temperature, III (blue)—holding at room temperature during 30 min (structural relaxation), IV (red)—heating up to 70 °C (*T_g_* + 5 °C).

The experiment was conducted for three specimens, and there were no notable differences in the obtained results affirming the excellent quality of the PU-SMP used in the study.

When the polymer is subjected to cold programming, the applied external force initially induces linear elastic deformation through the stretching of the polymer chains. Further loading allows the material to overcome the resistance to segmental rotation which is the first barrier against deformation. It includes yielding, leading to a subsequent increase in strain accompanied by a reduction in stress. Next, plastic deformation develops until the second barrier occurs. It impedes the alignment of chains in the direction of the applied load, resulting in an increase in stress as strain increases.

The molecular mechanisms responsible for strain fixity and recovery in the cold programming approach can be described as follows. During CP, the polymer chains or segments undergo deformation at a temperature below *T_g_*. Each chain or segment experiences strong or weak constraints, and some even have no constraints. Consequently, when the applied loading force is removed, unconstrained segments instantaneously return to their equilibrium position, leasing to immediate elastic spring back. Segments experiencing weaker constraints are in a non-equilibrium state and undergo some gradual recovery, resulting in a viscoelastic spring back. For those segments under strong constraints, the non-equilibrium configuration becomes fixed. The deformed chains or segments maintain equilibrium with the constraints, signifying an increase in internal energy. With the increase in temperature, a reduction in constraints occurs with the subsequent release of locked energy. Hence, the molecules or segments strongly constrained undergo a gradual transition from their non-equilibrium configuration to an equilibrium configuration, facilitating the shape recovery of a cold-programmed PU-SMP [41].

#### 3.3.2. Investigation of PU-SMP *T_g_* = 65 °C Shape Memory Properties at 45 °C

**Figure 9:**
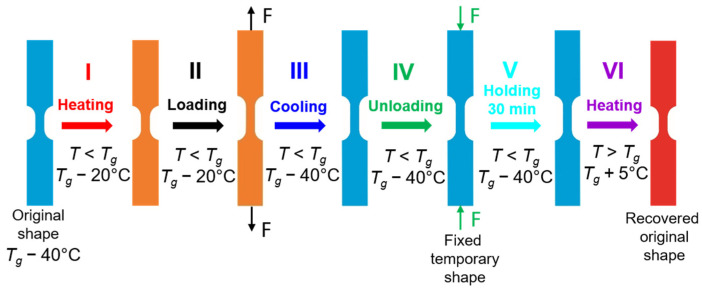
Schematic demonstration of the thermomechanical loading program at 45 °C presenting the subsequent stages of thermomechanical loading (I–VI) and showing the shape and gauge length changes in the PU-SMP specimen.

**Figure 10:**
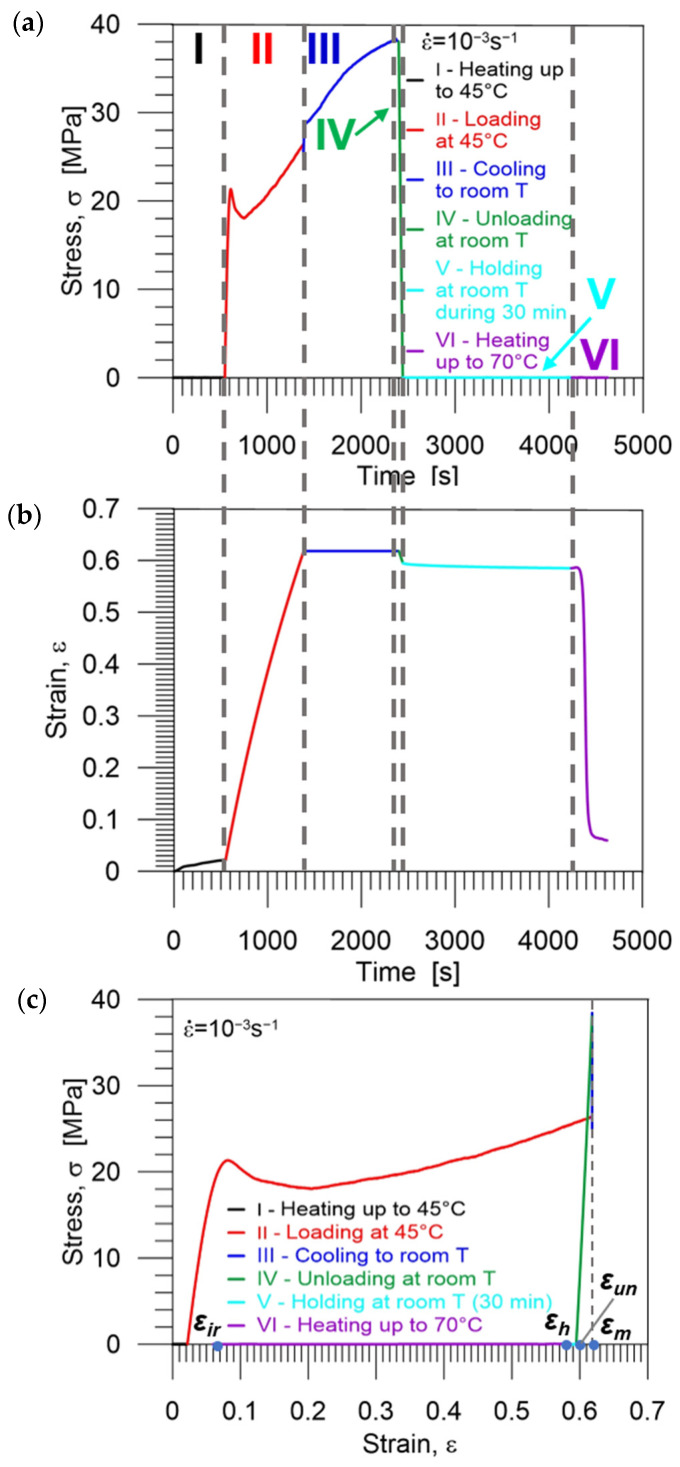
Stress (**a**) and strain (**b**) vs. time and (**c**) stress vs. strain curves obtained during the PU-SMP thermomechanical loading cycle at 45 °C. Particular colors mark subsequent stages of the program: I (red)—heating up to 45 °C, II (black)—loading at 45 °C, III (blue)—cooling down to room temperature, IV (green)—unloading at room temperature to zero stress, V (light blue)—holding at room temperature during 30 min, VI (violet)—second heating up to 70 °C (*T_g_* + 5 °C).

A complete shape memory cycle at 45 °C where the PU-SMP is deformed to a new shape and finally returned to its original shape is shown in Figure 9. In the first stage I, the specimen was heated up to a temperature of 45 °C (*T_g_* − 20 °C) at a heating rate of 12 °C/min. After that, the specimen was loaded by tension (stage II) with a strain rate of 10^−3^ s^−1^ at a temperature of 45 °C until the maximum strain of 60% (*ε_m_*) was obtained. Then, while maintaining the maximum strain value (*ε_m_*), the specimen was cooled down to room temperature 25 °C (*T_g_* − 40 °C) (stage III) in order to fix its temporary shape. Next, the specimen was unloaded (stage IV) to the zero-force value at 25 °C with a strain rate of 10^−3^ s^−1^. Some spring back occurred; however, the strain value obtained after unloading *ε_un_* was close to the maximum strain value *ε_m_*, showing the PU-SMP shape fixity property. The next stage (stage V) was a 30 min structural relaxation accompanied by viscoelastic spring back. Finally, the specimen was heated again from *T_room_* to *T_h_* = *T_g_ +* 5 °C = 70 °C at a heating rate of 12 °C/min under no-load conditions, leading to the recovery of the PU-SMP’s original shape (stage VI). The PU-SMP specimen recovered its shape; however, a residual strain *ε_ir_* was also recorded (Figure 10c). These experiments were also performed for three specimens, showing that the results are repeatable.

Diagrams demonstrating the procedure and showing the experimental results, obtained during the cycle of thermomechanical loading in the cold programming approach at 45 °C, i.e., the stress and strain vs. time as well as stress vs. strain curves are presented in Figure 10a, Figure 10b and Figure 10c, respectively. Selected colors used in the diagrams indicate each stage of the program: I (red)—heating up to 45 °C, II (black)—loading at 45 °C, III (blue)—cooling down to room temperature (25 °C), IV (green)—unloading at room temperature (25 °C) to zero stress, V (light blue)—holding at room temperature during 30 min, VI (violet)—second heating up to 70 °C. The strain values *ε_m_*, *ε_un_*, *ε_h_* and *ε_ir_* essential for the estimation of shape memory properties are marked by blue points in Figure 10c.

In Figure 7a, Figure 8b and Figure 10b,c, we observe that the strain decreases immediately upon unloading the PU-SMP due to spring back and structural relaxation in the case of cold programming. It is an undesirable process that diminishes PU-SMP functionality. During this step, the reorientation of polymer chains occurs. It should be noted that spring back is higher for the lower cold programming temperature which reduces shape fixity values.

#### 3.3.3. Investigation of PU-SMP Shape Memory Properties at *T_g_* = 65 °C

**Figure 11:**
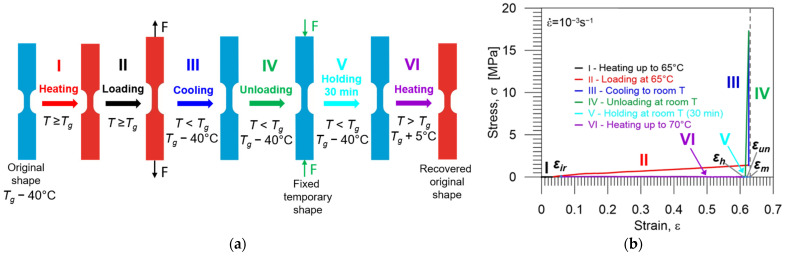
(**a**) Schematic demonstration of the thermomechanical loading program at 65 °C presenting the subsequent stages of thermomechanical loading (I–VI) and showing the shape and gauge length changes in the PU-SMP specimen; (**b**) stress vs. strain curve obtained during the PU-SMP thermomechanical loading cycle at 65 °C. Particular colors of curves mark subsequent stages of the program: I (red)—heating up to 65 °C, II (black)—loading at 65 °C, III (blue)—cooling down to room temperature, IV (green)—unloading at room temperature to zero stress, V (light blue)—holding at room temperature during 30 min, VI (violet)—second heating up to 70 °C (*T_g_* + 5 °C).

In order to ensure a possible wide spectrum of the experimental results and conduct comprehensive research, the shape memory properties have been also investigated in the conventional thermomechanical program, so-called hot programming, conducted at higher temperature—*T_g_* of the PU-SMP (65 °C) schematically illustrated in Figure 11a. In the first stage I, the specimen was heated to 65 °C (*T_g_*) at a rate of 12 °C/min. Following that, tension loading (stage II) was applied with a strain rate of 10^−3^ s^−1^ at 65 °C until reaching a maximum programmed strain of 60% (*ε_m_*). Then, the specimen was cooled to room temperature (25 °C) while maintaining the maximum strain (*ε_m_*) (stage III) to fix its temporary shape. Subsequently, the specimen was unloaded (stage IV) to zero force at 25 °C with a strain rate of 10^−3^ s^−1^, demonstrating the fixity of the new temporary shape. To maintain similar conditions for both cold programming and hot programming, the subsequent stage, which involved a 30 min holding at room temperature for relaxation (stage V), was also conducted. Finally, the specimen was reheated from room temperature to *T_h_* = 70 °C (*T_g_* + 5 °C) at a heating rate of 12 °C/min under no-load conditions, leading to the recovery of the PU-SMP’s original shape (stage VI). In order to confirm the repeatability of the results, these experiments were conducted on three specimens.

A diagram showing the experimental results obtained during the cycle of thermomechanical loading in a hot programming approach at 65 °C, i.e., the stress vs. strain, is presented in Figure 11b. Selected colors in the diagram indicate each stage of the program. The essential strain values (*ε_m_*, *ε_un_*, *ε_h_* and *ε_ir_*) crucial for determining shape memory properties are denoted by blue points.

It can be observed from Figure 11b that in the case of hot programming, the strain value obtained after unloading (*ε_un_*) is close to the maximum strain value *ε_m_*, demonstrating the excellent shape fixity property of the PU-SMP. Additionally, there are minimal differences between the strain value after unloading and the strain value after holding at room temperature, suggesting almost negligible spring back. Upon reheating to a temperature above *T_g_*, the PU-SMP specimen returns to its original shape; however, a residual strain (*ε_ir_*) was recorded, indicating incomplete total shape recovery.

Distinguishing from the cold programming approach, hot programming mechanisms for shape fixity and recovery are attributed to entropy elasticity or entropic force. It can be explained in the following way. During heating above *T_g_*, the soft segments in the polymer chains between the hard segments attain heightened flexibility, and rotations around the segment bonds become significantly enhanced. The system’s entropy increases, leading to higher numbers of macromolecular conformations, which, in turn, increases the ability of the polymer chains to turn into random coils. Even slight loads applied lead to the disentanglement of polymer chains, reducing their movements and causing a substantial decrease in entropy. Upon cooling the deformed polymer to a temperature below *T_g_*, the movement of polymer chains becomes constrained and the deformation is retained after the removal of constraints, resulting in the fixity of the temporary shape. Reheating the deformed polymer to a temperature above *T_g_* causes the chains to revert to random coils and restores the high entropy state. Therefore, the recovery of the original shape of the PU-SMP occurs [2,41].

#### 3.3.4. Determination and Comparison of Shape Fixity and Shape Recovery Parameters of PU-SMP *T_g_* = 65 °C Obtained in Various Conditions

Shape fixity and shape recovery parameters of PU-SMP with *T_g_* = 65 °C, essential for the PU-SMP applications, were determined during the cold programming approach at two temperatures (25 °C and 45 °C) and the results were compared to those obtained during the traditional hot programming approach conducted at temperature 65 °C.

The shape fixity *R_f_* and shape recovery *R_r_* parameters were calculated using the obtained experimental data (Figure 7, Figure 8, Figure 10 and Figure 11), according to Equations (1) and (2), proposed by H. Tobushi and S. Hayashi [19]:

(1) $$\;R_{f}=\frac{\varepsilon_{un}}{\varepsilon_{m}}\cdot 100\%,$$

(2) $$R_{r}=\frac{\varepsilon_{m}-\varepsilon_{ir}}{\varepsilon_{m}}\cdot 100\%,$$

where:

*ε_m_*—the maximum strain,
*ε_un_*—the strain obtained after unloading,
*ε_ir_*—the irrecoverable strain obtained after heating up to *T_g_* + 5 °C under no-load conditions.

Moreover, the values of shape fixity after holding for 30 min at room temperature were determined according to Equation (3) and added in Table 3:

(3) $$\;R_{fh}=\frac{\varepsilon_{h}}{\varepsilon_{m}}\cdot 100\%,$$

where:

*ε_h_*—the strain after holding for 30 min at room temperature.

The obtained PU-SMP shape fixity and shape recovery ratios determined for three specimens at each temperature are compared in Table 3. The results showed a non-significant discrepancy.

Shape fixity denotes the property of the shape memory polymers to maintain the programmed strain. The average shape fixity ratio of the PU-SMP at room temperature programming was found to be approximately 90%; the average shape fixity ratio at 45 °C was approximately 97%, while at *T_g_* = 65 °C, this parameter was equal to 98%. It means that the shape fixity is higher when the material is deformed at a higher temperature.

Shape fixity after holding indicates the degree of keeping a new shape after holding under no-load conditions. In the case of programming at room temperature, the average value of shape fixity after holding is equal to 83%, at 45 °C—95% and at 65 °C—97%, manifesting a higher amount of spring back and lower shape fixity after structural relaxation at a lower temperature.

The shape recovery parameter describes the ability of PU-SMP to restore the original shape which happens during heating to a temperature above *T_g_* when a deformed and frozen polymer chain becomes flexible and changes its configuration. The average shape recovery ratio was found to be 93% when programmed at room temperature of 25 °C and approximately 90% at 45 °C and at 65 °C, demonstrating that the shape recovery is higher during programming at a lower temperature. As can be noticed in Figure 10b,c and Figure 11b, during heating to 45 °C and 65 °C, a thermal elongation occurs, which acts against the shape recovery process [21]. Moreover, in the case of programming at 65 °C, the thermal elongation during the first heating process is higher than that obtained at 45 °C.

Generally, in hot programming, we observed larger shape fixity, shape fixity after holding and lower shape recovery values than in cold programming. However, when comparing values of these parameters obtained at 45 °C and 65 °C, the values of shape recovery are comparable and quite high at both temperatures, with slightly higher values of shape fixity and shape fixity after holding in the case of 65 °C programming. The values of shape fixity obtained in cold programming performed at room temperature are much lower than those obtained during programming at 45 °C and 65 °C due to spring back caused by structural relaxation, while the shape recovery values are higher.

However, it is worth noting that the shape recovery values at cold programming temperatures are rather high, which means that during heating above *T_g_,* the PU-SMP can effectively recover the plastic deformation and micro-damages that appeared as a result of loading at a temperature below *T_g_* beyond the yield limit. In the case of programming in room conditions, this allows for the avoidance of the heating and cooling steps to achieve a new shape at room temperature, which makes the PU-SMP attractive for various applications.

Although a thorough investigation has been conducted that broadens our understanding of the thermomechanical behavior of the PU-SMP and shape memory properties demonstrated in various conditions, future research is planned to explore other deformation modes and include a higher number of thermomechanical loading cycles.

## 4. Conclusions

The structural, mechanical and thermomechanical investigation of polyurethane shape memory polymers characterized by a glass transition temperature of 65 °C was conducted. The results of the identification of the functional properties in the cold programming approach in quite a large strain range significantly beyond the yield limit were obtained. The results were compared with those obtained in the hot programming approach.

An improved experimental procedure in the thermomechanical loading program was proposed which enabled the determination of the shape memory parameters with a higher accuracy since the three additional thermocouples mounted in the Instron thermal chamber assured significantly better control of the specimen temperature during the process.

The determined shape fixity ratio of the PU-SMP at room temperature programming (25 °C) was found to be approximately 90%, while the shape fixity ratio obtained at 45 °C (*T_g_* − 20 °C) was approximately 97%. Whereas, the shape recovery ratio was 93% at room temperature programming and it was equal to approximately 90% at 45 °C. The results obtained using traditional hot programming conducted at 65 °C indicate a higher shape fixity value (98%), but a lower shape recovery property of 90%. Thus, the obtained results confirmed good shape memory properties of the PU-SMPs at a large strain range at various temperatures.

The experiments conducted at both temperatures below *T_g_* demonstrated that the cold programming approach is effective and can be successfully applied to PU-SMPs with relatively high *T_g_*, without the risk of material degradation, caused by heating above *T_g_.* This confirmed the advantages of cold programming which allows us to save time and energy as well as to avoid the thermal expansion of the polymers, demonstrating at the same time, good shape memory properties.

Knowledge about PU-SMP shape memory and shape fixity properties estimated in cold programming makes them attractive for secure applications, especially important where heating above *T_g_* for reshaping is undesirable, e.g., in the case of applications in electronic components, aircraft or aerospace structures.

## Figures and Tables

**Table 1:** Crucial parameters obtained for PU-SMP with *T_g_* = 65 °C as the results of DMA analysis.

Storage Modulus *E*′ at 20 °C, MPa	*T_g_*, °C		*h*_tan *δ*_	Storage Modulus *E*′ at 80 °C, MPa
	*E’_onset_*	tan *δ*		
2160	50.17	65.21	1.17	4.42

**Table 2:** Mean values of mechanical properties of PU-SMP with *T_g_* = 65 °C obtained during tension with a strain rate of 10^−3^ s^−1^ until rupture at 25 °C, 45 °C and 65 °C.

Mechanical Property	At 25 °C	At 45 °C	At 65 °C
Young’s modulus, MPa	811.47 ± 3.36	513.67 ± 1.42	1.75 ± 0.05
Yield strength, MPa	53.01 ± 0.75	21.20 ± 0.23	−
Elongation at break, %	169.99 ± 2.78	187.30 ± 3.56	−

**Table 3:** The values of shape fixity and shape recovery parameters obtained for the PU-SMP with *T_g_* = 65 °C during the thermomechanical loading program using the cold programming approach.

Specimen	Loading Temperature of Cold-Programming, °C	Shape Fixity *R_f_,* %	Shape Holding *R_fh_,* %	Shape Recovery *R_r_,* %
1	25	89.25	83.26	93.06
2	25	89.47	82.64	92.96
3	25	89.88	84.04	92.98
Average	25	89.53 ± 0.32	83.31 ± 0.69	93.00 ± 0.05
1	45	96.81	95.33	89.87
2	45	96.57	95.05	90.28
3	45	96.21	94.63	90.38
Average	45	96.53 ± 0.30	95.00 ± 0.35	90.17 ± 0.27
1	65	97.97	97.37	90.42
2	65	98.34	97.48	90.31
3	65	98.14	96.01	90.19
Average	65	98.16 ± 0.18	96.95 ± 0.82	90.31 ± 0.12

## Data Availability

Data are contained within the article.
